# Treatment with A_2A_ receptor antagonist KW6002 and caffeine intake regulate microglia reactivity and protect retina against transient ischemic damage

**DOI:** 10.1038/cddis.2017.451

**Published:** 2017-10-05

**Authors:** Raquel Boia, Filipe Elvas, Maria H Madeira, Inês D Aires, Ana C Rodrigues-Neves, Pedro Tralhão, Eszter C Szabó, Younis Baqi, Christa E Müller, Ângelo R Tomé, Rodrigo A Cunha, António F Ambrósio, Ana R Santiago

**Affiliations:** 1 Institute for Biomedical Imaging and Life Sciences (IBILI), Faculty of Medicine, University of Coimbra, Coimbra 3000-548, Portugal; 2 CNC.IBILI, University of Coimbra, Coimbra, Portugal; 3 Center for Neuroscience and Cell Biology (CNC), Faculty of Medicine, University of Coimbra, Coimbra 3004-504, Portugal; 4 Department of Chemistry, Faculty of Science, Sultan Qaboos University, 123 Muscat, Oman; 5 Pharmazeutische Chemie I, Pharmazeutisches Institut, University of Bonn, Bonn, Germany; 6 Faculty of Medicine, University of Coimbra, Coimbra 3004-504, Portugal; 7 Association for Innovation and Biomedical Research on Light and Image (AIBILI), Coimbra 3000-548, Portugal

## Abstract

Transient retinal ischemia is a major complication of retinal degenerative diseases and contributes to visual impairment and blindness. Evidences indicate that microglia-mediated neuroinflammation has a key role in the neurodegenerative process, prompting the hypothesis that the control of microglia reactivity may afford neuroprotection to the retina against the damage induced by ischemia–reperfusion (I–R). The available therapeutic strategies for retinal degenerative diseases have limited potential, but the blockade of adenosine A_2A_ receptor (A_2A_R) emerges as candidate strategy. Therefore, we evaluated the therapeutic potential of a selective A_2A_R antagonist (KW6002) against the damage elicited by I–R. The administration of KW6002 after I–R injury reduced microglia reactivity and inflammatory response and afforded protection to the retina. Moreover, we tested the ability of caffeine, an adenosine receptor antagonist, in mediating protection to the retina in the I–R injury model. We demonstrated that caffeine administration dually regulated microglia reactivity and cell death in the transient retinal ischemic model, depending on the reperfusion time. At 24 h of reperfusion, caffeine increased microglial reactivity, inflammatory response and cell death elicited by I–R. However, at 7 days of reperfusion, caffeine administration decreased microglia reactivity and reduced the levels of proinflammatory cytokines and cell death. Together, these results provide a novel evidence for the use of adenosine A_2A_R antagonists as potential therapy for retinal ischemic diseases and demonstrate the effect of caffeine on the regulation of microglia-mediated neuroinflammation in the transient ischemic model.

Transient retinal ischemia refers to a pathological condition that involves loss of blood supply to the tissue, resulting in energy depletion, dysfunction, damage and death of neuronal cells.^1^ This feature determines the pathophysiology of several retinal diseases like acute closed-angle glaucoma and diabetic retinopathy, contributing to visual impairment and blindness. Currently, there is no cure for these retinal diseases and the available treatments are not very effective, being of particular interest to identify novel therapeutic strategies to manage these disorders.

The model of acute elevation of intraocular pressure (IOP) followed by reperfusion (ischemia–reperfusion, I–R) has been used to study molecular mechanisms underlying retinal ischemia and to devise new potential therapeutic strategies.^2^ Microglial cells, the immunocompetent cells of the central nervous system (CNS) and the first responders to neuronal injury,^3, 4^ become reactive upon retinal I–R,^5^ as occurs in retinal degenerative diseases.^8^ Sustained microglia activation leads to excessive production of inflammatory mediators that contribute to retinal neurodegeneration.^8, 9^ This prompts the possibility that systems able to control microglia reactivity might be suitable to manage the neurodegenerative process.

One candidate strategy is operated by the adenosinergic system, namely the ability of the adenosine A_2A_ receptor (A_2A_R) blockade in controlling microglia reactivity, thus affording neuroprotection.^10, 11, 12^ Recently, we demonstrated that a selective A_2A_R antagonist (SCH58261) prevents retinal microglia reactivity and neuroinflammation elicited by elevated pressure in an *in vitro* model.^13^ Moreover, intravitreal administration of SCH58261 prior I–R injury prevents microglia-mediated neuroinflammation and confers protection to the retina.^5^ However, it is still unknown the effects of A_2A_R antagonist administered after I–R.

Caffeine is the most widely consumed psychoactive drug and at nontoxic doses targets the adenosine receptors, mainly the inhibitory adenosine A_1_ receptor (A_1_R) and the facilitatory A_2A_R.^14^ Caffeine has been demonstrated to afford robust neuroprotection under different neurotoxic situations in the brain, an effect that is mediated by the blockade of A_2A_R.^15, 16, 17, 18, 19^ Moreover, we found that caffeine attenuated the loss of retinal ganglion cells (RGCs) in animals with ocular hypertension.^20^ Still, it remains unknown whether caffeine protects against retinal I–R injury as this is a pathophysiological process contributing to cellular damage in multiple ocular conditions.

The aims of this work were to investigate the therapeutic potential of oral administration of A_2A_R antagonist and the effects of caffeine intake against neuroinflammation and cell death triggered by I–R injury.

## Results

### Blockade of A_2A_R prevented proinflammatory response in retina triggered by transient ischemia

**Figure 1:**
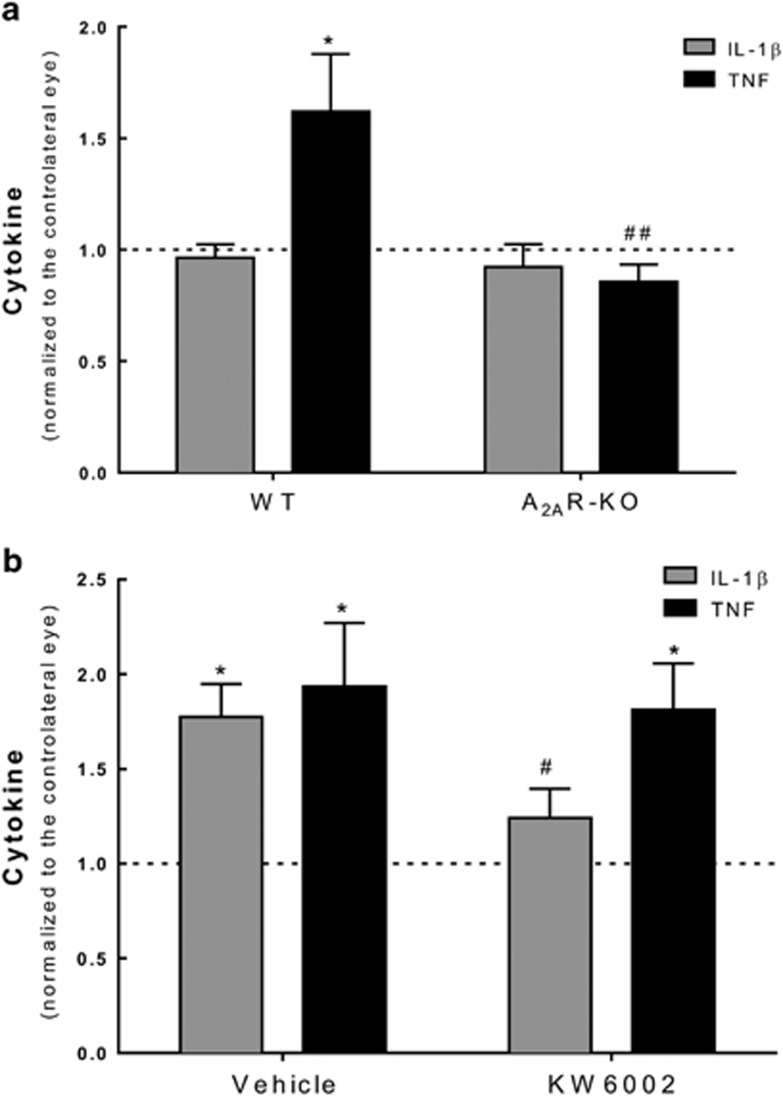
(**a**) Effects of genetic blockade of A_2A_R in the levels of IL-1*β* and TNF in the retina subjected to transient ischemia. A_2A_R-KO mice were subjected to ischemic injury. The protein levels of IL-1*β* and TNF were quantified by ELISA at 7 days of reperfusion. Results are expressed as the ratio of ischemic eye compared with contralateral eye. *P<0.05, significantly different from contralateral eye, Wilcoxon Signed-Rank test; ^##^*P*<0.01 significantly different from vehicle-treated animals, Student’s *t*-test. (**b**) Effects of treatment with KW6002 in the protein levels of IL-1*β* and TNF upon transient ischemia. KW6002 (3 mg/kg) was administered by oral gavage 2 h post injury and until the end of experiment (7 days of reperfusion). The levels of IL-1*β* and TNF were quantified by ELISA. Results are expressed as the ratio of ischemic eye compared with contralateral eye. *P<0.05, significantly different from contralateral eye, Wilcoxon's signed-rank test; ^#^*P*<0.05 significantly different from vehicle-treated animals, Student’s *t-*test

Recently, we demonstrated that A_2A_R antagonist prevents RGC death through the control of microglia-mediated neuroinflammation.^5, 13^ Therefore, we evaluated whether A_2A_R-knockout (KO) animals were less prone to neuroinflammation triggered by ischemic damage in the retina. The levels of proinflammatory cytokines tumor necrosis factor (TNF) and interleukin-1*β* (IL-1*β*) were quantified in the retinas of A_2A_R-KO and WT animals (Figure 1a). The induction of ocular hypertension unilaterally has been described to trigger activation of microglia in the contralateral eye.^21^ Having this in account, the results obtained in I–R retinas were normalized to contralateral eye. Retinal I–R did not significantly change IL-1*β* levels in both groups of animals (A_2A_R-KO and WT). However, TNF levels were significantly reduced in I–R-subjected A_2A_R-KO retinas (I–R/contralateral ratio of 0.9±0.08, *n*=6, *P*<0.01) compared with WT animals (I–R/contralateral ratio of 1.6±0.3, *n*=6, *P*<0.05).

Taking these and other results^5, 13^ into account, we further extended the study to assess the therapeutic potential of A_2A_R antagonist against retinal I–R injury. Herein, the treatment with a selective A_2A_R antagonist (KW6002, istradefylline) started after I–R injury and was given daily for 7 days, as opposed to our previous work in which a single intravitreal injection of A_2A_R antagonist was given prior I–R.^5^ IL-1*β* and TNF protein levels were quantified in the retina by ELISA (Figure 1b). In the I–R retinas of vehicle-treated animals, IL-1*β* and TNF protein levels were 1.8±0.2- (*n*=8, *P*<0.05) and 1.9±0.3-fold (*n*=8, *P*<0.05) above the contralateral retinas, respectively. In KW6002-treated rats, IL-1*β* protein levels in I–R retinas were not significantly different from contralateral retinas (1.2±0.2-fold change, *n*=8) and were significantly reduced compared with vehicle-treated animals (*P*<0.05). TNF levels increased by 1.8±0.2-fold in I–R retinas compared with contralateral retinas (*n*=9, *P*<0.05) in KW6002-treated animals.

### Treatment with KW6002 reduced microglia reactivity upon I–R injury

**Figure 2:**
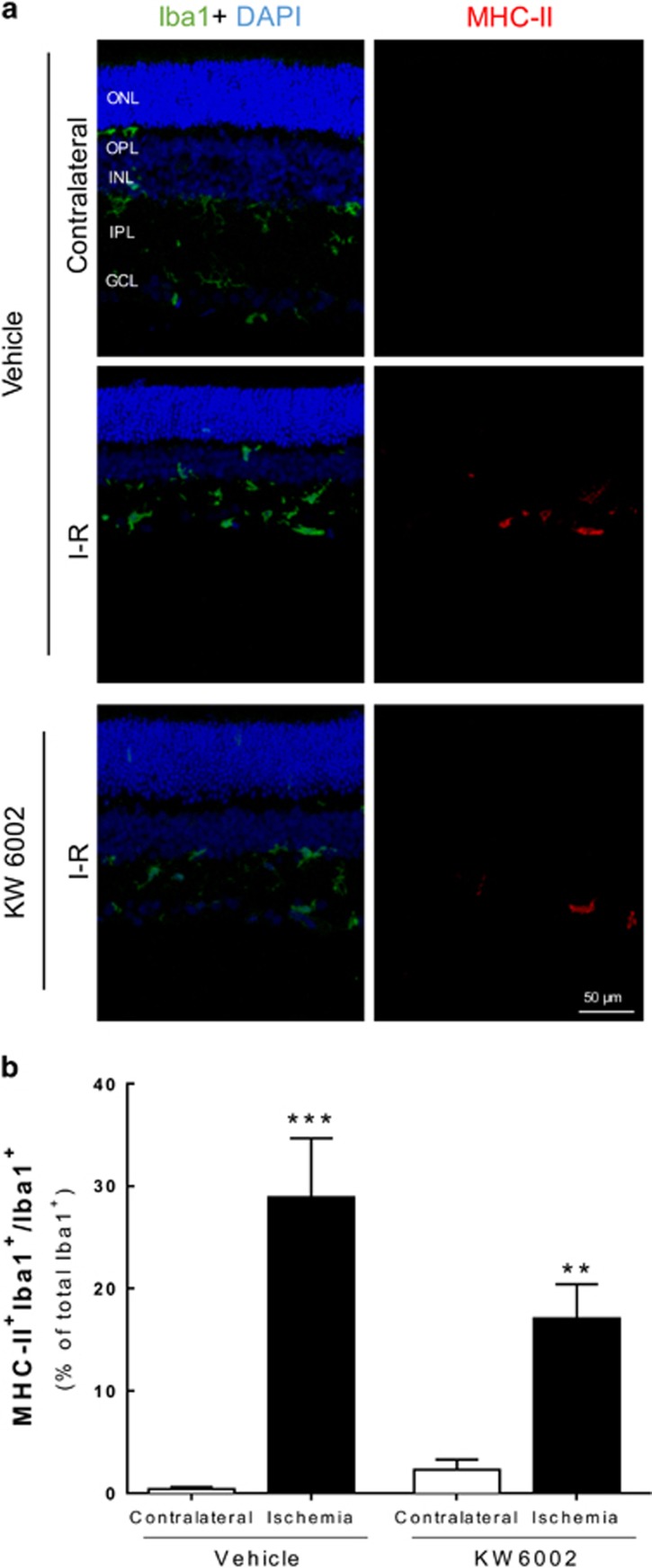
Effects of treatment with KW6002 in microglia reactivity elicited by transient ischemia. KW6002 (3 mg/kg) was administered by oral gavage 2 h post injury and until the end of experiment (7 days of reperfusion). (**a**) Microglia reactivity was assessed in retinal sections by labeling with major MHC-II (reactive microglia, red) and Iba1 (general marker, green) at 7 days (**a**) of reperfusion. Nuclei were stained with DAPI (blue). Representative images are depicted. (**b**) The number of activated microglia/macrophages (MHC-II^+^Iba1^+^ cells) was expressed as the percentage of total number of microglia/macrophages (Iba1^+^ cells) in the retinal section at 7 days of reperfusion. **P<0.01, ***P<0.001, significantly different from contralateral eye, Mann–Whitney test. ONL, outer nuclear layer; OPL, outer plexiform layer; INL, inner nuclear layer; IPL, inner plexiform layer; GCL, ganglion cell layer

The effect of KW6002 on retinal I–R-induced microglia reactivity was assessed by counting the number of cells immunoreactive to both major histocompatibility complex class II (MHC-II; expressed in reactive microglia) and ionized calcium-binding adaptor molecule 1 (Iba1; constitutively expressed in microglia) (Figure 2 and Supplementary Figure S1). Microglia reactivity (MHCII^+^Iba1^+^/Iba1^+^ cells) was significantly increased in I–R retinas (28.9±5.8% of total Iba1^+^ cells, *n*=8, *P*<0.001) compared with the contralateral retinas from vehicle-treated animals (0.4±0.2% of total Iba1^+^ cells, *n*=7). KW6002 treatment attenuated microglia reactivity (17.1±3.3% of total Iba1^+^ cells, *n*=8, *P*<0.01) elicited by I–R, whereas no significant changes were observed in the contralateral retinas (2.3±1.0% of total Iba1^+^ cells, *n*=8) compared with contralateral retinas of vehicle-treated animals. Additionally, KW6002 treatment (49.5±3.5 Iba1^+^ cells, *n*=2) reduced the increased number of Iba1^+^ cells induced by retinal I–R (119.0±18.2 Iba1^+^ cells, *n*=3) (Supplementary Figure S1).

### KW6002 reduces cell death induced by transient retinal ischemia

**Figure 3:**
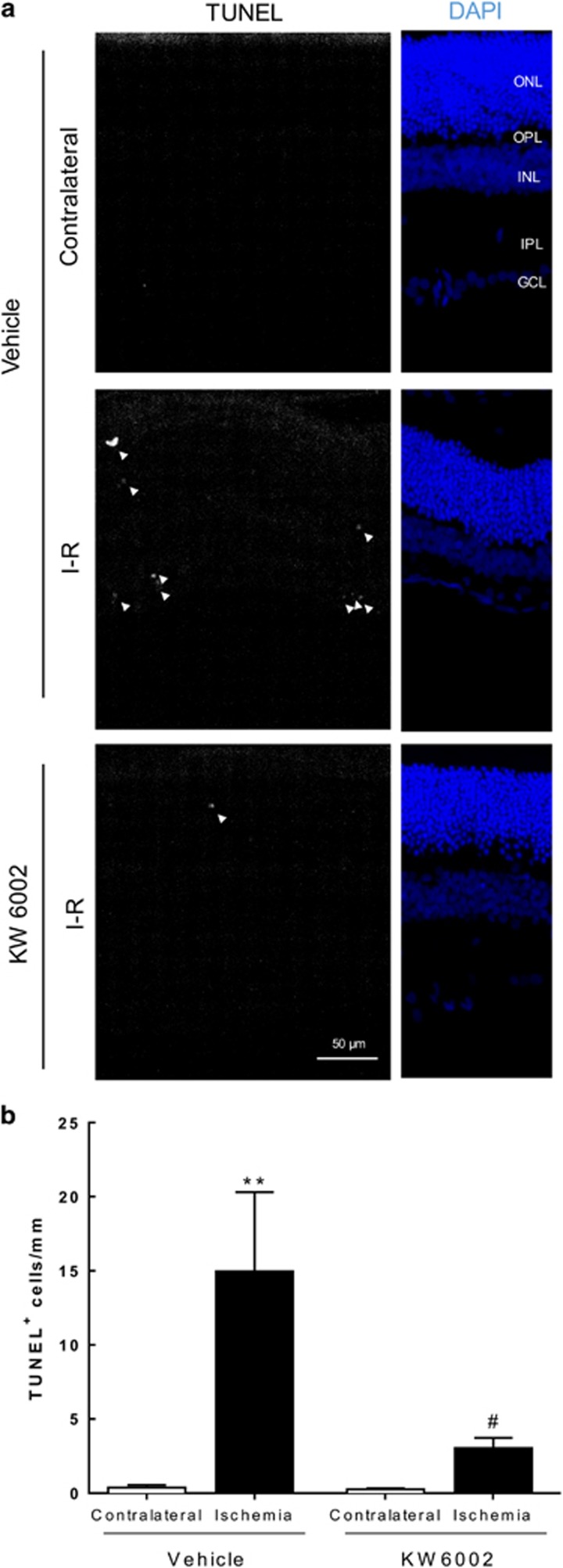
Effects of KW6002 treatment in retinal cell death upon I–R injury. KW6002 (3 mg/kg) was administered by oral gavage 2 h post injury and until the end of experiment (7 days of reperfusion). (**a**) Cell death was assayed in retinal cryosections by TUNEL assay at 7 days of reperfusion. Nuclei were stained with DAPI (blue) and representative images are depicted. (**b**) TUNEL^+^ cells (gray, some TUNEL^+^ cells are indicated with arrowheads) were counted and expressed per mm of retina. **P<0.01, significantly different from contralateral eye; ^#^*P*<0.05, significantly different from I–R retinas of KW6002-treated animals, Mann–Whitney test. ONL, outer nuclear layer; OPL, outer plexiform layer; INL, inner nuclear layer; IPL, inner plexiform layer; GCL, ganglion cell layer

As transient retinal ischemia elicits neuronal cell death,^22^ and neuroinflammation may contribute to neurodegeneration,^5^ we assessed the protective effect of KW6002 treatment by counting cell death with the terminal deoxynucleotidyl transferase-mediated dUTP nick-end labeling (TUNEL) assay (Figure 3). In vehicle-treated animals, I–R significantly increased the number of retinal apoptotic cells (15.0±5.4 TUNEL^+^ cells per mm, *n*=7, *P*<0.01) compared with contralateral retinas (0.4±0.2 TUNEL^+^ cells per mm, *n*=6). The KW6002 treatment significantly decreased I–R-triggered cell death (3.0±0.7 TUNEL^+^ cells per mm, *n*=6, *P*<0.05) compared with vehicle-treated I–R retinas. The number of apoptotic cells in the contralateral retinas of animals treated with KW6002 (0.3±0.1 TUNEL^+^ cells per mm, *n*=6) was not significantly different from the vehicle-treated animals. These results show that A_2A_R blockade has therapeutic potential against damage induced by transient retinal ischemia.

### Caffeine modulates the expression and levels of proinflammatory cytokines

Caffeine (1 g/l) was administered in the drinking water for 2 weeks before I–R and until the end of the experiment (24 h or 7 days of reperfusion). The fluid intake was not statistically different between the two groups of animals (Table 1), as reported previously.^23^ The consumption of caffeine and the concentration of caffeine in serum and retinal samples (obtained immediately after killing the animals) are shown in Table 1. Some reports demonstrated that caffeine can influence IOP,^24^ an effect that was not observed in this study (Table 1).

Taking into consideration the protective properties of A_2A_R blockade against retinal injury, and as caffeine is an adenosine receptor antagonist that has demonstrated to afford protection in CNS noxious conditions, including the retina,^20^ we assessed the effects of caffeine intake in I–R injury. Despite that the protective potential of KW6002 was assessed at 7 days of reperfusion, our previous work demonstrated the preventive effect of A_2A_R antagonist at 24 h of reperfusion.^5^ Therefore, we studied the effects of caffeine intake against I–R damage at 24 h and 7 days of reperfusion.

**Figure 4:**
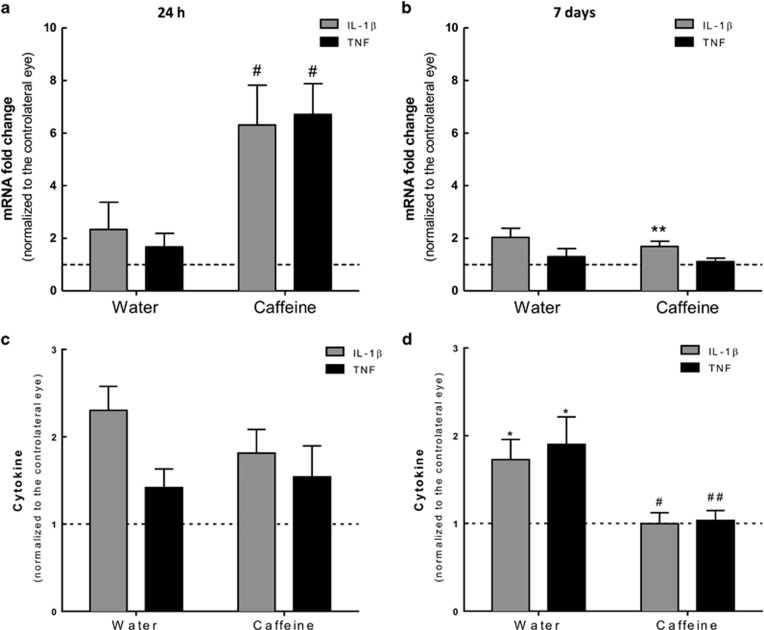
Effects of caffeine administration in the expression and release of IL-1*β* and TNF elicited by transient ischemia. Caffeine (1 g/l) was administered in the drinking water for 2 weeks prior injury and until the end of the experiment (24 h and 7 days of reperfusion). (**a** and **b**) The mRNA expression of IL-1*β* and TNF was assessed by qPCR at 24 h (**a**) and 7 days (**b**) of reperfusion. Results are presented as fold change of ischemic eye to contralateral eyes. **P<0.01, significantly different from contralateral eye, Wilcoxon's signed-rank test; ^#^*P*<0.05 significantly different from I–R retinas of water-drinking animals, Mann–Whitney test. (**c** and **d**) The protein levels of IL-1*β* and TNF were quantified by ELISA at 24 h (**c**) and 7 days (**d**) of reperfusion. Results are expressed as the ratio of ischemic eye to contralateral eye. *P<0.05, significantly different from contralateral eye, Wilcoxon's signed-rank test; ^#^*P*<0.05, ^##^*P*<0.01, significantly different from I–R retinas of water-drinking animals, Mann–Whitney test

Then, the effect of caffeine administration in the mRNA expression and protein levels of IL-1*β* and TNF were evaluated by quantitative PCR (qPCR) and ELISA, respectively (Figure 4).

At both 24 h and 7 days of reperfusion, IL-1*β* and TNF transcript levels were not statistically different in I–R retinas compared with contralateral retinas in water-drinking animals (*P*>0.05) (Figures 4a and b). However, at 24 h of reperfusion, the caffeine-drinking I–R group presented significantly increased IL-1*β* and TNF transcripts levels, compared with water-drinking I–R injured retinas (6.3±1.5- and 6.7±1.2-fold above contralateral, *n*=5 and *n*=5, for IL-1*β* and TNF, respectively, *P*<0.05). The analysis of mRNA expression of IL-1*β* and TNF of caffeine-drinking animals at 7 days of reperfusion demonstrated an increase of IL-1*β* (1.7±0.2-fold above contralateral, *n*=10, *P*<0.01), without significant changes in the TNF expression.

Concerning IL-1*β* and TNF protein levels (Figures 4c and d), at 24 h of reperfusion, I–R retinas of the water-drinking animals, revealed a 2.3±0.3-fold above the contralateral retinas of IL-1*β* levels (*n*=5, *P*=0.06), while only a modest increase was detected in TNF levels (I–R/contralateral ratio of 1.4±0.2, *n*=5, *P*=0.06). Caffeine administration did not significantly alter IL-1*β* and TNF levels compared with water-drinking animals (*n*=6 and *n*=7, *P*=0.06 and *P*=0.22 for IL-1*β* and TNF, respectively). At 7 days of reperfusion, IL-1*β* and TNF protein levels in the water-drinking animals I–R retinas were still higher than the contralateral retinas (I–R/contralateral ratio of 1.7±0.2 and 1.9±0.3 for IL-1*β* and TNF, *n*=6 and *n*=9, respectively, *P*<0.05). Notably, caffeine administration reduced this effect (I–R/contralateral ratio of 1.0±0.1, *n*=13, *P*<0.05 and 1.0±0.1, *n*=12, *P*<0.01 for IL-1*β* and TNF, respectively).

### Caffeine has a dual regulation of microglia reactivity induced by transient ischemia

**Figure 5:**
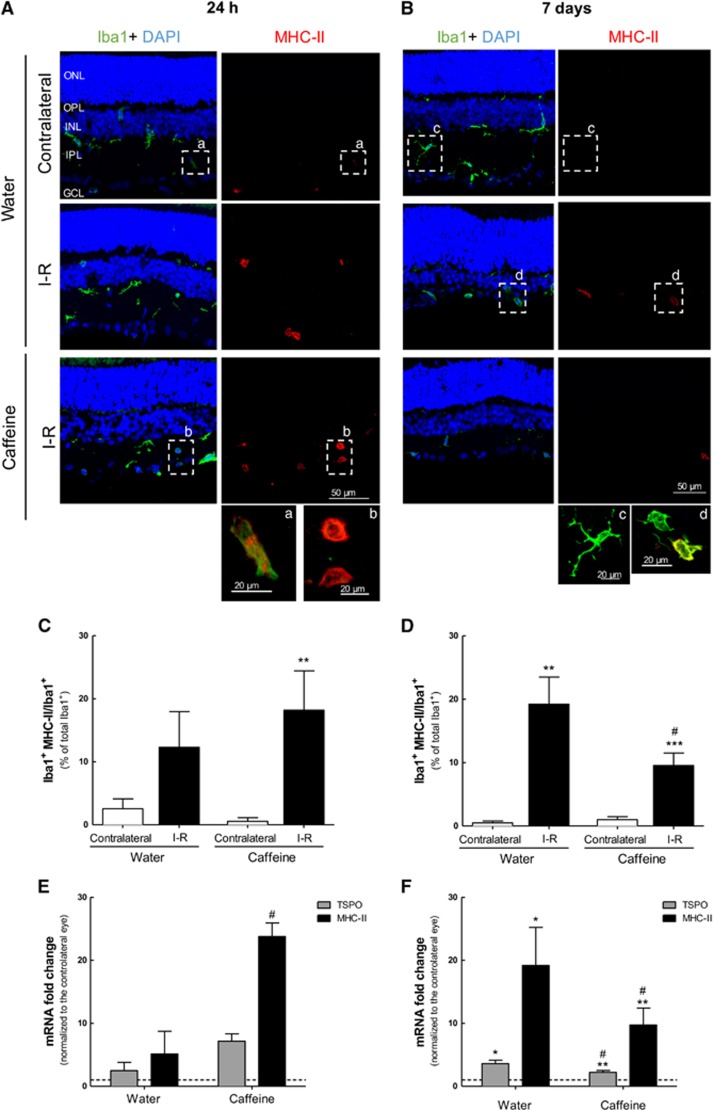
Effect of caffeine administration in microglia reactivity elicited by I–R injury. Caffeine (1 g/l) was administered in the drinking water for 2 weeks prior ischemic injury and until the end of the experiment (24 h and 7 days of reperfusion). (**A** and **B**) Microglia reactivity was assessed in retinal sections by labeling with major MHC-II (reactive microglia, red) and Iba1 (general marker, green) at 24 h (a) and 7 days (b) of reperfusion. Nuclei were stained with DAPI (blue). Representative images are depicted. Note in the inserts (a–d) the alteration of microglia morphology from ramified to ameboid morphology, indicating microglia reactivity. (**C** and **D**) The number of reactive microglia/macrophages (MHC-II^+^Iba1^+^ cells) was expressed as the percentage of total number of microglia/macrophages (Iba1^+^ cells) in the retinal section at 24 h (c) and 7 days (d) of reperfusion. **P<0.01, ***P<0.001, significantly different from contralateral eye; ^#^*P*<0.05, significantly different from I–R retinas of water-drinking animals, Mann–Whitney test. (**E** and **F**) The mRNA expression of the markers of microglia activation, TSPO and MHC-II, was assessed by qPCR after 24 h (e) and 7 days (f) of reperfusion. Results are presented as fold change of the contralateral eye. *P<0.05, **P<0.01, significantly different from contralateral eye, Wilcoxon's signed-rank test; ^#^*P*<0.05, significantly different from water-drinking group, Mann–Whitney test. ONL, outer nuclear layer; OPL, outer plexiform layer; INL, inner nuclear layer; IPL, inner plexiform layer; GCL, ganglion cell layer

The effect of caffeine on microglia reactivity triggered by ischemia followed by 24 h or 7 days of reperfusion was analyzed by immunohistochemical staining of retinal sections (Iba1 and MHC-II, as described) and by mRNA expression with qPCR (Figure 5 and Supplementary Figures S2 and S3). As expected, transient retinal ischemia triggered an increase in microglia reactivity (Figures 5a–d and Supplementary Figures S2 and S3), also observed by the transition from ramified to amoeboid morphology (Figure 5, inserts a–d). At 24 h after reperfusion (Figure 5c and Supplementary Figure S2), microglia reactivity was slightly increased in I–R retinas (12.3±5.7% of total Iba1^+^ cells, *n*=5) compared with the contralateral retinas (2.5±1.6% of total Iba1^+^ cells, *n*=5). In caffeine-drinking rats, microglia reactivity was significantly increased in I–R retinas (18.2±6.2% of total Iba1^+^ cells, *n*=8, *P*<0.01) compared with the contralateral retina (0.6±0.6% of total Iba1^+^ cells, *n*=8). Moreover, at 24 h of reperfusion, caffeine administration increased the number of Iba1^+^ cells (48.0±7.0 Iba1^+^ cells, *n*=2) compared with water-drinking I–R animals (68.0±5.5 Iba1^+^ cells, *n*=3) (Supplementary Figure S2).

At 7 days of reperfusion (Figure 5d and Supplementary Figure S3), microglia reactivity was significantly increased in I–R retinas of water-drinking animals (19.2±4.3% of total Iba1^+^ cells, *n*=6, *P*<0.01) compared with the contralateral retina (0.5±0.3% of total Iba1^+^ cells, *n*=6). Caffeine significantly attenuated microglia reactivity elicited by transient ischemia (9.6±2.0% of total Iba1^+^ cells, *n*=11, *P*<0.05) compared with I–R retinas from water-drinking animals (1.0±0.5% of total Iba1+ cells, *n*=11). Additionally, after 7 days of reperfusion, caffeine administration decreased the number of Iba1^+^ cells induced by I–R (132.7±35.9 Iba1^+^ cells, *n*=3) compared with water-drinking group (89.3±3.8 Iba1^+^ cells, *n*=3) (Supplementary Figure S3).

The increase in mRNAs coding for translocator protein (18 kDa) (TSPO) and for the MHC-II family (Cd74) has been used as marker of microglia reactivity.^5, 25^ Therefore, we investigated whether caffeine changed the mRNA levels of microglia-related proinflammatory markers MHC-II and TSPO elicited by transient retinal ischemia (Figures 5e and f). In the water-drinking animals, the two markers were upregulated in the retina following I–R, reaching statistically significance at 7 days of reperfusion (3.6±0.5- and 19.2±6.1-fold above contralateral eye, *n*=6 and *n*=7, for TSPO and MHC-II, respectively, *P*<0.05). Caffeine intake differentially regulated the expression of the markers of reactive microglia depending on the reperfusion time. Caffeine administration enhanced the expression levels of TSPO and MHC-II at 24 h of reperfusion. However, at 7 days of reperfusion, the expression levels of TSPO and MHC-II in the retina in animals drinking caffeine significantly decreased, compared with water-drinking animals (2.2±0.3- and 9.7±2.7-fold above contralateral, *n*=10 and *n*=10, for TSPO and MHC-II, respectively, *P*<0.05).

### Caffeine regulates cell death induced by transient retinal ischemia

**Figure 6:**
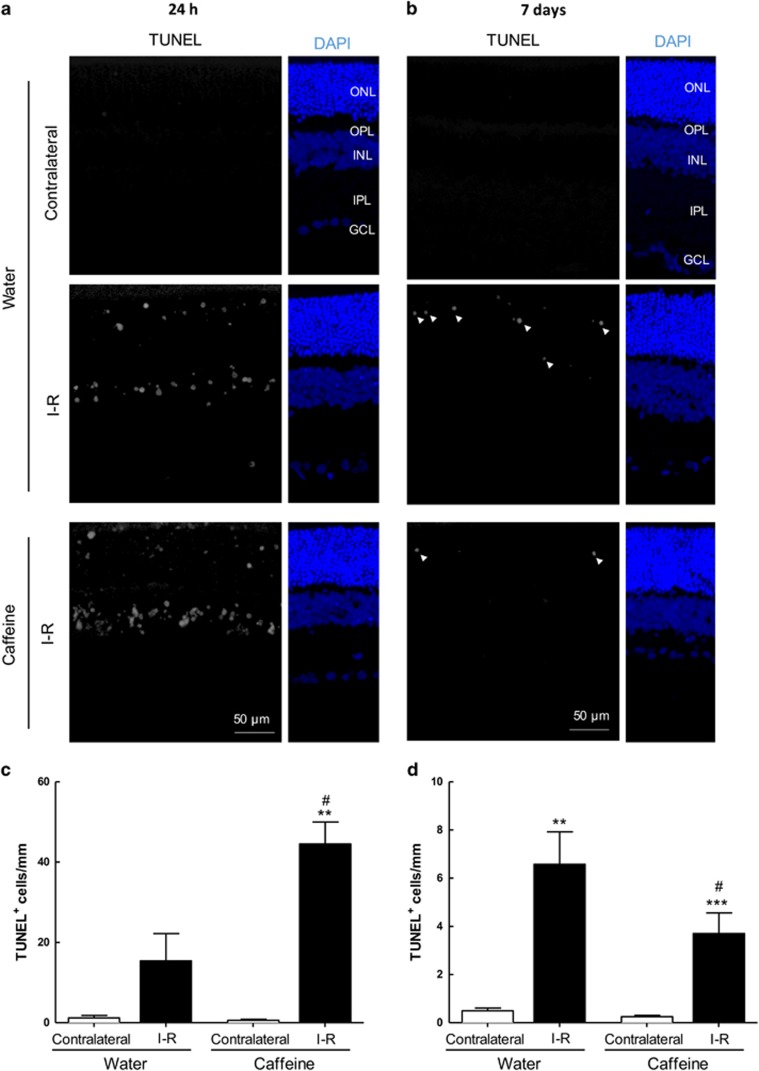
Effects of caffeine administration in cell death induced by transient retinal ischemia. Caffeine (1 g/l) was administered in the drinking water for 2 weeks prior injury and until the end of the experiment (24 h and 7 days of reperfusion). (**a** and **b**) Cell death was assayed in retinal cryosections by TUNEL assay at 24 h (**a**) and 7 days (**b**) of reperfusion. Nuclei were stained with DAPI (blue). Representative images are depicted. (**c** and **d**) TUNEL^+^ cells (gray, some TUNEL^+^ cells are indicated with arrowheads) were counted and were expressed per mm of retina. **P<0.01 and ***P<0.001, significantly different from contralateral eye; ^#^*P*<0.05, significantly different from I–R retinas of water-drinking animals, Mann–Whitney test. ONL, outer nuclear layer; OPL, outer plexiform layer; INL, inner nuclear layer; IPL, inner plexiform layer; GCL, ganglion cell layer

We finally assessed the effects of caffeine on apoptotic cell death at 24 h and 7 days of reperfusion (Figure 6).

Cell death is an early event in the transient retinal ischemia model, as observed by the presence of TUNEL^+^ cells already at 24 h of reperfusion (15.4±6.8 TUNEL^+^ cells per mm, *n*=4), significantly higher than the contralateral retinas (1.2±0.6 TUNEL^+^ cells per mm, *n*=5) of water-drinking animals (Figure 6c). Caffeine administration increased the number of apoptotic cells after 24 h (44.5±5.5 TUNEL^+^ cells per mm, *n*=4, *P*<0.01) compared with contralateral retinas (0.6±0.3 TUNEL^+^ cells per mm, *n*=6). Importantly, there were no significant differences (*P*>0.05) in the number of TUNEL^+^ in contralateral retinas (Figure 6c) and administration of caffeine to naïve animals (non-ischemic animals drinking caffeine for 2 weeks) did not cause significant alterations in the number of apoptotic cells (0.6±0.4 TUNEL^+^ cells per mm, *n*=4, data not shown).

At 7 days of reperfusion (Figure 6d), the number of apoptotic cells in I–R retinas of water-drinking animals was increased (6.6±1.3 TUNEL^+^ cells per mm, *n*=5, *P*<0.01) compared with contralateral retinas (0.5±0.1 TUNEL^+^ cells per mm, *n*=5). Also, caffeine significantly reduced the number of apoptotic cells in I–R retinas (3.7±0.9 TUNEL^+^ cells per mm, *n*=10, *P*<0.05) compared with contralateral retinas (0.3±0.1 TUNEL^+^ cells per mm, *n*=10). Administration of caffeine for 3 weeks did not change the number of TUNEL^+^ cells in non-ischemic retinas (0.17±0.02 TUNEL^+^ cells per mm, *n*=2, data not shown).

## Discussion

This study demonstrates that oral treatment with KW6002, a selective A_2A_R antagonist, initiated after ischemia, effectively reduces neuroinflammation and affords protection to the retina. Moreover, we demonstrate that caffeine administration dually regulates microglia reactivity and cell death in the transient retinal ischemic model, depending on the reperfusion time: it initially exacerbates microglia reactivity and increases neuronal death, whereas it affords a sustained and time-delayed protection of microglia reactivity and neuroprotection in the retina.

Transient ischemia induced by high IOP represents a useful model to study the histopathological changes in the retina. Retinal I–R injury model recapitulates several features of retinal ischemic diseases, including neuronal death, inflammation and microglia reactivity.^5, 7, 26^ The ischemic period directly triggers loss of retinal cellular functions,^27^ whereas the reperfusion of the retinal tissue triggers additional injury, probably because of oxidative stress and generation of free radicals.^1^ Therefore, retinal response to I–R involves a dual process of damage, which is dependent both on the duration and magnitude of ischemia, as well as on the time of reperfusion.^28^

Blockade of A_2A_R directly controls neuroinflammation^12^ and affords robust protection in different neurodegenerative diseases associated with chronic neuroinflammation.^29, 30^ We and others have demonstrated that A_2A_R blockade prevents retinal cell death and microglia-mediated neuroinflammation in different *in vitro* and animal models of retinal degenerative diseases, including the retinal I–R injury rat model.^5, 13, 31, 32^ Recently, we reported that a single intravitreal injection of SCH58261, a selective A_2A_R antagonist, prior the induction of retinal ischemia, prevents microglia reactivity and cell loss elicited by I–R (24 h of reperfusion).^5^

In retinal I–R model, IL-1*β* and TNF seem to be the major effectors of retinal damage.^33, 34^ As the deletion of A_2A_R does not induce changes in the levels of TNF in the retina,^35^ the reduced levels of TNF in A_2A_R-KO mice after transient retinal ischemia advances the prospects that A_2A_R depletion is able to reduce inflammation upon retinal injury.

Despite the fact that genetic deletion or pharmacological blockade of A_2A_R afford protection to the retina, the therapeutic effect of A_2A_R antagonist was not known. Hence, we further extended the study of assessing the therapeutic potential of A_2A_R antagonist against retinal I–R injury. KW6002 (istradefylline) is considered the most suitable A_2A_R antagonist for oral administration in CNS studies, based on its bioavailability, half-life and brain penetration.^36^ Additionally, KW6002 has undergone a clinical trial for Parkinson’s disease as its safety has been established,^37^ being approved for the adjunctive treatment of Parkinson’s disease in Japan.^38^

The administration of KW6002 after inducing ischemia protected the retina against transient ischemic damage. Although both IL-1*β* and TNF were increased in ischemic retinas and are known to mediate I–R damage,^34^ KW6002 only reduced the levels of IL-1*β*, suggesting that it might be sufficient to induce cell loss. In previous work, we reported that intravitreal administration of SCH58261 prevents the increase in IL-1*β* without affecting TNF.^5^ It has been suggested that the neuroprotection afforded by A_2A_R blockade might result from the ability of A_2A_R to control IL-1*β*-induced exacerbation of excitotoxic neuronal damage.^39, 40^ Although we cannot identify the cell types involved in the proinflammatory response, microglial cells are the main contributors for the increase in the inflammatory response.^8^ Additionally, our previous works support the contention that pretreatment with A_2A_R antagonist in microglia hampers microglia-mediated neuroinflammation and affords protection to the retina.^5, 13^ Accordingly, we now observed that KW6002 also decreased microglia reactivity, probably through a direct action on microglia as these cells are endowed with A_2A_Rs and their expression increase under noxious conditions.^5, 13, 31^ Indeed, previous works have been demonstrating protective properties of A_2A_R antagonists administered by intravitreal injection^5, 31^ and intraperitoneal injection.^16^ To our knowledge, this is the first report demonstrating that the oral administration of a selective A_2A_R antagonist has therapeutic properties against retinal damage. This may be of particular relevance in a clinical setting as the treatment of chronic retinal neurodegenerative diseases would require multiple intravitreal injections, posing additional complications, as infections or retinal detachment.^41^

The effects of caffeine are operated by the blockade of adenosine receptors, mainly A_2A_R and A_1_R.^14^ In fact, A_2A_R seems to be the main target of caffeine, when mediating neuroprotection.^15, 16, 17, 18, 19^ We previously found that caffeine attenuated the loss of RGC in animals with ocular hypertension.^20^ Although the protective results with KW6002 were only assessed at 7 days of reperfusion, our previous work demonstrated the preventive effects of A_2A_R antagonist at 24 h of reperfusion.^5^ Therefore, we designed our study to assess the effects of caffeine intake against retinal I–R injury at both 24 h and 7 days of reperfusion. We found that the effects of caffeine depend on the time of reperfusion analyzed.

At 24 h of reperfusion, caffeine increased the mRNA encoding for IL-1*β* and TNF, without alterations in their protein content; by contrast, at 7 days of reperfusion, caffeine significantly decreased the protein levels of IL-1*β* and TNF. Moreover, we found that caffeine intake differently impacts microglia reactivity depending on the reperfusion time, inhibiting microglia activation at later time points upon injury (7 days of reperfusion). Notably, the results in the retina recapitulate the apparent short- and long-term effects of caffeine on microglia reactivity in the brain parenchyma at different time periods after application of different insults.^10, 42, 43^ Caffeine administration has been reported to decrease the number of reactive microglia in the brain parenchyma upon prolonged exposure to 6-hydroxydopamine^42^ or chronic infusion of lipopolysaccharide (LPS).^10^ In contrast, caffeine potentiates striatal activation of microglia and astroglia elicited by 3,4-methylenedioxymethamphetamine (MDMA) administration,^43^ although this was only evaluated 48 h after MDMA administration, leaving open the possibility that caffeine differentially controls microglia reactivity at later time points. Moreover, this further prompts microglia as the main contributors for the increase of inflammatory response,^8^ as heralded by the reported ability of caffeine to suppress LPS-induced proinflammatory mediators, as nitric oxide (NO), prostaglandin E2 and TNF in murine BV-2 microglial cell line.^44^

Increased microglia reactivity and release of inflammatory mediators can lead to activation of apoptotic pathways that contribute to the pathogenesis of retinal I–R injury.^45^ Apoptotic cell death is an early event in I–R injury model, being detected in the three nuclear layers.^46^ At 24 h of reperfusion, apoptotic cell death was more evident in the inner nuclear layer (INL), whereas at 7 days of reperfusion, most of the cells undergoing apoptosis were located in the outer nuclear layer (ONL). Several reports show that consumption of moderate doses of caffeine affords protection against noxious conditions (reviewed in Gomes *et al.*^30^). The present work revealed that caffeine actually has a biphasic impact on the control of cell death in the retina. At 24 h of reperfusion, caffeine increased cell death associated with I–R, whereas at 7 days of reperfusion caffeine afforded protection to the retina. This biphasic effect of caffeine was also noted in an animal model of multiple sclerosis: caffeine affords protection only in the effector phase of the disease, when degeneration occurs, and not in the initial phase of the disease, corresponding to the initial inflammation.^47^ Taking these observations into consideration, one might speculate that this biphasic effect of caffeine might be related to a particular impact of caffeine on signaling pathways in microglia that are important for the resolution of chronic inflammation. Specific patterns of gene expression occur in the retina due to ischemia and at different time points of reperfusion.^28^ The time-dependent effects of caffeine likely reflect the different modulation by caffeine on the distinct signaling events or cellular processes during reperfusion to ultimately confer protection to the retina against I–R injury. Moreover, we cannot discard the effects of caffeine metabolites in the retina. As caffeine is rapidly metabolized to theophylline and paraxanthine (the main metabolites in rodents within 120 min^14^), it is possible that these metabolites may also contribute to neuroprotection, as described for paraxanthine in models of Parkinson’s disease^48, 49^ and theobromine in models of Alzheimer’s disease.^50^

In summary, this work demonstrates that the effect of caffeine administration in the rat model of retinal transient ischemia is dependent of the time of reperfusion. Importantly, at the later time point assessed (7 days of reperfusion), the administration of caffeine afforded protection to the retina against transient ischemic damage, suggesting that exacerbated microglia reactivity at the earlier time point is critical in the resolution of inflammation and the chronic intake of caffeine is beneficial to the retina. We suggest that future studies on potential neuroprotective drug targets should take into consideration several time-points. Moreover, we also demonstrated for the first time that a selective A_2A_R antagonist (KW6002) administered orally has therapeutic interest against transient retinal ischemic damage, suggesting that A_2A_R antagonist can be further studied to manage retinal ischemic diseases or neurodegenerative diseases.

## Materials and methods

### Animals

Wistar rats and wild-type (WT) and global A_2A_R-KO C57BL/6 mice were housed under controlled environment (temperature of 21.8±0.1 °C of temperature and 67.6±1.6% relative humidity, 12 h light/12 h dark cycle) with free access to food and water. All procedures involving animals were approved by the Animal Welfare Committee of the Faculty of Medicine of University of Coimbra and were conducted in accordance with the European Community directive guidelines for the use of animals in laboratory (2010/63/EU), transposed into the Portuguese law in 2013 (Decreto-Lei 113/2013) and were in agreement with the Association for Research in Vision and Ophthalmology statement for animal use.

### Measurement of IOP

The IOP was measured bilaterally 3 days a week with a rebound tonometer specifically designed for rodents (Tonolab; Icare, Vantaa, Finland) between 1:00 p.m. and 4:00 p.m. Six reliable measurements were made in each eye and an internal software generated an average value after elimination of high and low readings. For the purpose of this study, the generated average was considered as one reading and reported as the IOP for that eye. Basal IOP values were taken in the week before starting the administration of caffeine.

### Drug administration

**Figure 7:**
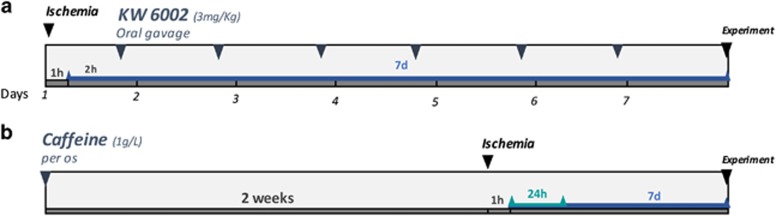
Schematic overview of the rat experimental design. (**a**) Treatment with KW6002 (3 mg/kg) started (orally by gavage) 2 h after retinal ischemia (60 min), and once a day until the end of the experiment (7 days of reperfusion). The green line (**a**) or the blue line (**a** and **b**) indicate the reperfusion time. (**b**) Caffeine (1 g/l) was administered in the drinking water for 2 weeks prior induction of ischemia (60 min duration) and until the end of the experiment (24 h or 7 days of reperfusion)

For the study testing the therapeutic potential of selective A_2A_R antagonist, the rats were treated with KW6002 (3 mg/kg) by oral gavage, 2 h after the induction of I–R and daily until the end of the experiment (Figure 7a). The animals were randomly divided into animals treated with KW6002 (istradefylline, 3 mg/kg in vehicle solution; synthesized as previously described^51^) or animals that were given vehicle solution (0.025% methylcellulose). The study involving caffeine administration was performed using a concentration (1 g/l) previously reported to afford neuroprotection upon CNS injury.^18, 23, 52^ Caffeine (1 g/l; Sigma-Aldrich, St. Louis, MO, USA) was supplied in the drinking water for 2 weeks before the induction of ischemia and until the end of the experiment (Figure 7b). The solution of caffeine was prepared fresh every 2 days. The animals were randomly divided into drinking water or drinking caffeine.

### Retinal transient ischemic injury (I–R)

Retinal I–R injury was performed as we described previously.^5^ Briefly, animals were anesthetized with 2.5% isoflurane (IsoFlo; Abbott Laboratories, Chicago, USA) in 1 l/min O_2_, and placed on a heating plate to maintain their body temperature throughout the ischemic procedure. After topical anesthesia with oxybuprocaine (4 mg/ml, Anestocil, Edol) and pupillary dilation with tropicamide (10 mg/ml, Tropicil Top, Edol), the anterior chamber of one eye was cannulated with a 30-gauge needle (rats) or with a 33-gauge needle (mice) connected to a reservoir infusing sterile saline solution. Pressure in the eye was increased to 80 mm Hg (measured with Tonolab) for 60 min (rats) or 45 min (mice) and the contralateral eye was considered as control. To avoid corneal opacity, viscoelastic solution (2% Methocel; Dávi II-Farmacêutica SA, Barcarena, Portugal) was applied to both eyes. After 60 min of ischemia the needle was withdrawn and reperfusion was established. Fusidic acid ointment (10 mg/g, Fucithalmic; Leo Pharmaceutical, Ballerup, Denmark) was applied at the end of the experiment to prevent infection. Animals drinking caffeine (1 g/l) were killed at 24 h or 7 days of reperfusion, and animals treated with KW6002 (3 mg/kg), WT and A_2A_R-KO mice were killed at 7 days of reperfusion.

### Quantification of caffeine levels

In the serum, caffeine was quantified by high-performance liquid chromatography using a reverse-phase column (LiChro-CART 125 × 4 mm^2^ LiChrospher 100 RP-18 (5 *μ*m) cartridge fitted into a ManuCART holder; Merck, Darmstadt, Germany) and a Gilson system equipped with a UV detector set at 274 nm, as described previously.^52^ Serum samples were obtained after centrifugation of blood at 2000 × *g* for 15 min.

In the retina, caffeine concentration was determined by using a caffeine/pentoxifylline Enzyme-Linked Immunosorbent Assay (ELISA) Kit (Neogen Corporation, Lansing, MI, USA), following the instructions provided by the manufacturer with some modifications, as follows. The retinas were homogenized in enzyme immunoassay buffer (EIA) provided with the kit and then were sonicated and centrifuged at 10 000 × *g*, at 4 °C for 5 min, and the supernatant was collected. The concentration of caffeine/pentoxifylline was determined using a standard curve prepared with a caffeine solution (0–600 ng/ml in EIA).

### Immunohistochemistry

Animals were deeply anesthetized with an intraperitoneal injection of a solution of ketamine (90 mg/kg; Imalgene 1000) and xylazine (10 mg/kg; Ronpum 2%) and then transcardially perfused with phosphate-buffered saline (PBS, in mM: 137 NaCl, 2.7 KCl, 10 Na_2_HPO_4_, 1.8 KH_2_PO_4_; pH 7.4), followed by 4% (w/v) paraformaldehyde (PFA). The eyes were enucleated and postfixed in 4% PFA for 1 h. Then, the cornea was carefully dissected out and the eyecup was fixed for an additional 1 h in 4% PFA. After washing in PBS, the tissue was cryopreserved in 15% sucrose in PBS for 1 h, followed by 30% sucrose in PBS for 1 h. The eyecups were embedded in tissue-freezing medium (optimal cutting temperature (oct); Shandon Cryomatrix (Thermo Scientific, Waltham, MA, USA)) with 30% of sucrose in PBS (1 : 1), and stored at −80 °C. The tissue was sectioned on a cryostat (Leica CM3050 S, Leica Biosystems, Wetzlar, Germany) into 10 *μ*m thickness sections and mounted on Superfrost Plus glass slides (Menzel-Gläser; Thermo Scientific).

For immunohistochemistry, retinal sections were fixed with ice-cold acetone at −20 °C for 10 min, and then rehydrated in PBS two times until the removal of OCT. The tissue was permeabilized with 0.25% Triton X-100 in PBS for 30 min. The sections were blocked in 10% normal goat serum plus 1% bovine serum albumin (BSA) in PBS for 30 min at room temperature in a humidified environment. After washing with PBS, the sections were incubated overnight with the primary antibody (Table 2) prepared in 1% BSA in PBS at 4 °C, in a humidified environment. Then, the sections were rinsed with PBS followed by incubation with the corresponding secondary antibodies (Table 2), prepared in 1% BSA in PBS for 1 h at room temperature, in the dark. The sections were washed with PBS and then incubated with the nuclear dye 4',6-diamidino-2-phenylindole (DAPI), diluted 1 : 2000. The tissue was washed in PBS and mounted with Glycergel mounting medium.

### TUNEL assay

Cell death was detected with a TUNEL Assay Kit (Promega Corporation, Madison, WI, USA) with fluorescein detection following the instructions provided by the manufacturer. The nuclei were counterstained with DAPI (1 : 2000). Sections were washed in PBS and mounted with Glycergel mounting medium.

### Image analysis

For the analysis of microglia reactivity, the preparations were observed with a confocal microscope (LSM 710; Zeiss, Oberkochen, Germany) on an Axio Observer Z1 using a Plan ApoChromat x20/0.8 objective. From each eye, four sections were used and six images per section were acquired. In each image, the number of cells immunoreactive to both MHC-II and Iba1 (MHC-II^+^Iba1^+^) was counted and expressed as a percentage of the total number of cells immunoreactive to Iba1 (Iba1^+^). Representative images were acquired using a EC Plan-Neofluar x40/1.30 Oil DIC M27 objective. Z-stacks images were acquired and merged using the maximum intensity projection mode of the Zeiss Software (Zen 2009; Zeiss).

For the quantification of cell death (TUNEL staining), the preparations were observed in a fluorescence microscope (Axio observer Z1), using a LD Plan-Neofluar x40/0.6 Korr Ph2 M27 objective. From each eye, four sections were analyzed and the number of TUNEL^+^ cells was counted in the entire retinal section and normalized to the length of the respective section. Representative images were acquired with a confocal microscope (LSM 710; Zeiss) on an Axio Observer Z1 using a EC Plan-Neofluar x40/1.30 Oil DIC M27 objective. Z-stacks images were acquired as described above.

### Real-time qPCR

Total RNA was extracted from rat retinas using Trizol reagent (Invitrogen, Life Technologies, Carlsbad, CA, USA). RNA samples were dissolved in 16 *μ*l of RNase-free water. Total RNA concentration was determined using a NanoDrop ND1000 (Thermo Scientific, Waltham, MA, USA). The quality of total RNA was determined (2100 Bioanalyser). The integrity of RNA, expressed as the RNA integrity number, was between 7.7 and 9.6, indicating high-quality, non-degraded RNA.

The amplification of cDNA from 1 *μ*g of total RNA was performed according to the instructions provided by the manufacturer (NZYTech, Lisbon, Portugal). The resultant cDNA was treated with RNAse-H for 20 min at 37 °C, and a 1 : 2 dilution was prepared for real-time qPCR analysis. All samples were stored at −20 °C until analysis.

Genomic DNA contamination was assessed with a conventional PCR for *β*-actin using intron-spanning primers (Table 3), as described previously.^53^ SYBR-Green-based qPCR was performed using StepOnePlus (Applied Biosystems, Foster City, CA, USA), as described previously,^5^ with the following conditions: iTaq Universal SYBR-Green Supermix (Bio-Rad, Hercules, CA, USA), 200 nM primers (Table 3) and 2 *μ*l of 1:2 dilution cDNA, in a total volume of 20 *μ*l. Three candidate housekeeping genes (*Hprt1*, *Ywhaz* and *Rho*) were evaluated using NormFinder (a Microsoft Excel Add-in), and *Hprt1* was the most stable gene, and was used as the control gene. Ct values were converted to 'Relative quantification' using the 2^−ΔΔCt^ method described previously.^54^

### Quantification of TNF and IL-1*β* protein levels by ELISA

Protein levels of IL-1*β* and TNF in the retinas from Wistar rats, WT and A_2A_R-KO mice were quantified using ELISA, according to the instructions provided by the manufacturer (Peprotech, Rocky Hill, CT, USA).

The retinas were dissected in ice-cold PBS, and then were homogenized in 20 mM imidazole-HCl, 100 mM KCl, 1 mM MgCl_2_, 1 mM EGTA, 1 mM EDTA, 1% Triton X-100, supplemented with complete mini protease inhibitor cocktail tablets (Roche, Basel, Switzerland) and phosphatase inhibitors (10 mM NaF and 1 mM Na_3_VO_4_). Then, samples were sonicated and centrifuged at 10 000 × *g* for 5 min at 4 °C. The supernatant was collected and stored at −80 °C until use. The protein concentration of each sample was determined by the bicinchoninic acid protein assay according to the manufacturer’s instructions (Pierce Biotechnology, Waltham, MA, USA). The cytokine concentration of each sample was normalized to the total protein concentration. The results represent the ratio between the I–R-injured retina and the contralateral eye.

### Statistical analysis

The results are presented as mean±standard error of the mean. Statistical analysis was performed with the Prism 5.03 Software for Windows (GraphPad Software Inc., La Jolla, CA, USA). The normality of the data was assessed with Shapiro–Wilks and Kolmogorov–Smirnov normality tests. Accordingly, data were analyzed with nonparametric or parametric tests, as indicated in the figure legends. For the qPCR and ELISA analysis, the statistical differences between I–R and contralateral retinas were evaluated using a Wilcoxon's signed-rank test. Values of *P*<0.05 were considered statistically significant.

## Publisher’s Note:

Springer Nature remains neutral with regard to jurisdictional claims in published maps and institutional affiliations.

## Figures and Tables

**Table 1:** Parameters evaluated in animals involved in the study

	**Water-drinking rats**			**Caffeine-drinking rats**		
	**Basal**	**2 weeks treatment**	**3 weeks treatment**	**Basal**	**2 weeks treatment**	**3 weeks treatment**
Fluid intake (ml per day)		36±3	31±4		30±2	28±1
Caffeine consumption (mg/kg per day)					125±7	110±9
Serum caffeine (*μ*M)					54.6±20.5	97.5±10.7
Retinal caffeine/pentoxifylline (ng/mg prot)					277±25	189±26
IOP (mmHg)	11±0.2	12±0.5	12±0.8	12±0.4	12±0.3	12±1.0

Abbreviation: IOP, intraocular pressure.

**Table 2:** List of primary and secondary antibodies used in this study

	**Supplier**	**Cat. no.**	**Host**	**Dilution**
*Primary antibodies*				
Anti-Iba1	Wako (Richmond, VA, USA)	019-19741	Rabbit	1 : 1000
Anti-MHC class II	AbD Serotec (Hercules, CA, USA)	MCA46R	Mouse	1 : 200
				
*Secondary antibodies*				
Alexa Fluor anti-rabbit 488	Life Technologies (Carlsbad, CA, USA)	A11008	Goat	1 : 200
Alexa Fluor anti-mouse 568	Life Technologies	A11004	Goat	1 : 200

**Table 3:** Primers for quantitative PCR analysis

**Gene**	**GeneBank number**	**Forward**	**Reverse**	**Amplicon size (bp)**
*Actb*	NM_031144.2	5′-GCTCCTCCTGAGCGCAAG-3′	3′-CATCTGCTGGAAGGTGGACA-5′	75
*Hprt1*	XM_003752155	5′-ATGGGAGGCCATCACATTGT-3′	3′-ATGTAATCCAGCAGGTCAGCAA-5′	77
*Rho*	NM_033441.1	5′-GCAACAGGAGTCGGCTACCA-3′	3′-GCATAGGGAAGCCAGCAGATC-5′	76
*Ywhaz*	NM_013011.3	5′-CAAGCATACCAAGAAGCATTTGA-3′	3′-GGGCCAGACCCAGTCTGA-5′	76
*Tnf*	NM_012675	5′-CCCAATCTGTGTCCTTCT-3′	3′-TTCTGAGCATCGTAGTTGT-5′	90
*il1b*	NM_031512	5′-ATAGAAGTCAAGACCAAAGTG-3′	3′-GACCATTGCTGTTTCCTAG-5′	109
*Cd74*	NM_013069.2	5′-CCACCTAAAGAGCCACTGGA-3′	3′-AGAGCTGGCTTCTGTCTTCAC-5′	101
*Tspo*	NM_012515.2	5′-TGTATTCGGCCATGGGGTATG-3′	5′-GAGCCAGCTGACCAGTGTAG-3′	105
