# Supplementary material for: Treatment with A2A receptor antagonist KW6002 and caffeine intake regulate microglia reactivity and protect retina against transient ischemic damage
Source: Cell Death Dis. 2017 Oct 5;8(10):e3065–. doi: 10.1038/cddis.2017.451 (PMC5680573; doi:10.1038/cddis.2017.451)
Supplement: Supplementary Figures [file cddis2017451x1.pdf]

Treatment with A_{2A} receptor antagonist KW6002 and caffeine intake regulate microglia reactivity and protect retina against transient ischemic damage

Raquel Boia[1,2], Filipe Elvas[1], Maria H. Madeira[1,2], Inês D. Aires[1,2], Catarina Neves[1,2], Pedro Tralhão[1], Eszter C. Szabó[3], Younis Baqi[4], Christa E. Müller[5], Ângelo R. Tomé[2,3], Rodrigo A. Cunha[2,6], António F. Ambrósio[1,2,7], Ana R. Santiago[1,2,7*]

[1]Institute for Biomedical Imaging and Life Sciences (IBILI), Faculty of Medicine, University of Coimbra, 3000-548 Coimbra, Portugal; [2]CNC.IBILI, University of Coimbra, Portugal; [3]Center for Neuroscience and Cell Biology (CNC), Faculty of Medicine, University of Coimbra, 3004-504 Coimbra, Portugal; [4]Department of Chemistry, Faculty of Science, Sultan Qaboos University, 123 Muscat, Oman; [5]Pharmazeutische Chemie I, Pharmazeutisches Institut, University of Bonn, Germany; [6]Faculty of Medicine, University of Coimbra, 3004-504 Coimbra, Portugal; [7]Association for Innovation and Biomedical Research on Light and Image (AIBILI), 3000-548 Coimbra, Coimbra, Portugal

Supplementary Figure 1 – Retina whole mounts stained with anti-Iba1 in KW6002-treated animals

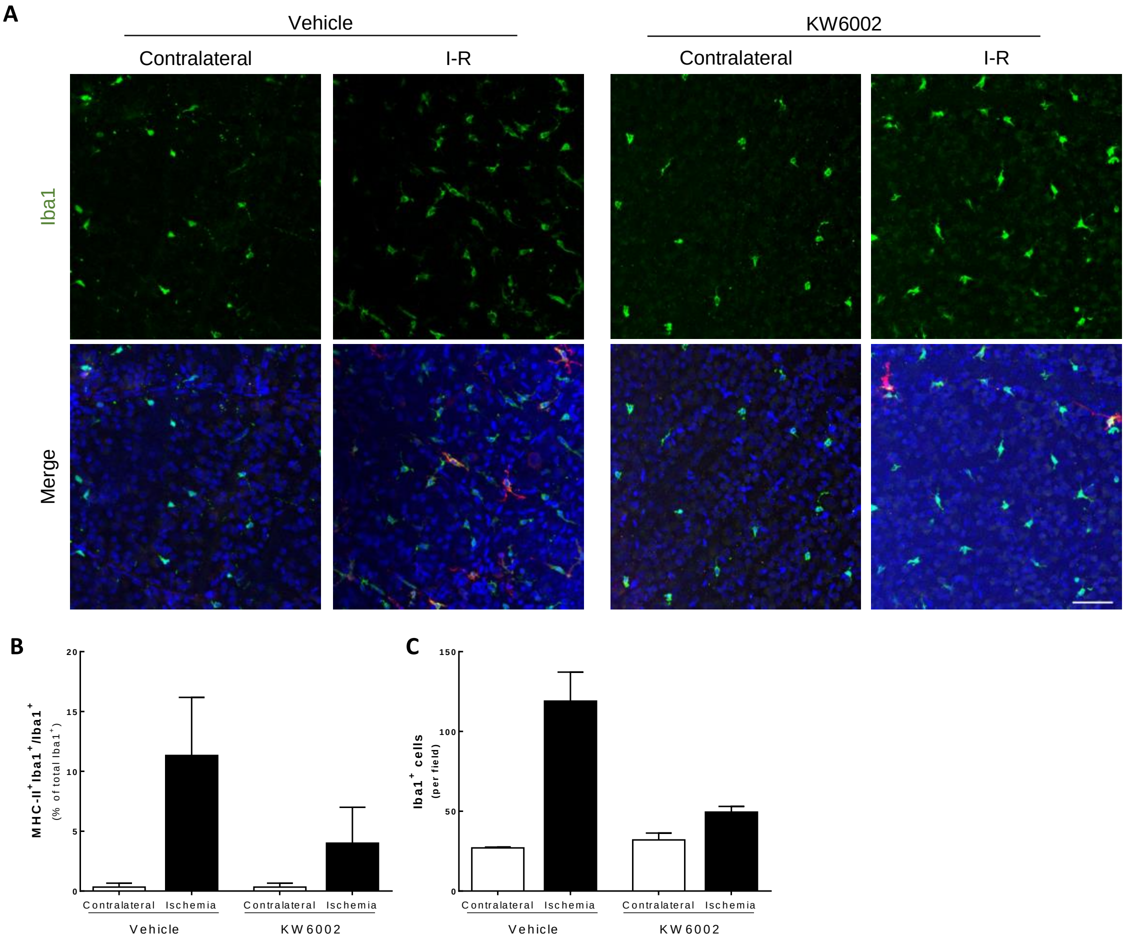

Supplementary Figure S1 - Effect of KW6002 treatment **(A, B, C)** in microglia reactivity elicited by transient ischemia. **A:** Microglia reactivity was assessed in retinal whole mounts by labeling with major histocompatibility complex class II (MHC-II, reactive microglia, red) and ionized calcium-binding adaptor molecule 1 (Iba1, general marker, green) at 7 days of reperfusion. Nuclei were stained with DAPI (blue). Representative images for each condition are depicted. **B:** The number of activated microglia/macrophages (MHC-II$^+$Iba1$^+$ cells) was counted and expressed as percentage of total number of microglia/macrophages (Iba1$^+$ cells) in the retinal whole mounts at 7 days of reperfusion. **C:** The number of microglia/macrophages (Iba1$^+$ cells) was counted in retinal whole mounts at 7 days of reperfusion. Scale bar: 50 µm.

Supplementary Figure 2 - Retina whole mounts stained with anti-Iba1 in animals drinking caffeine at 24h of reperfusion

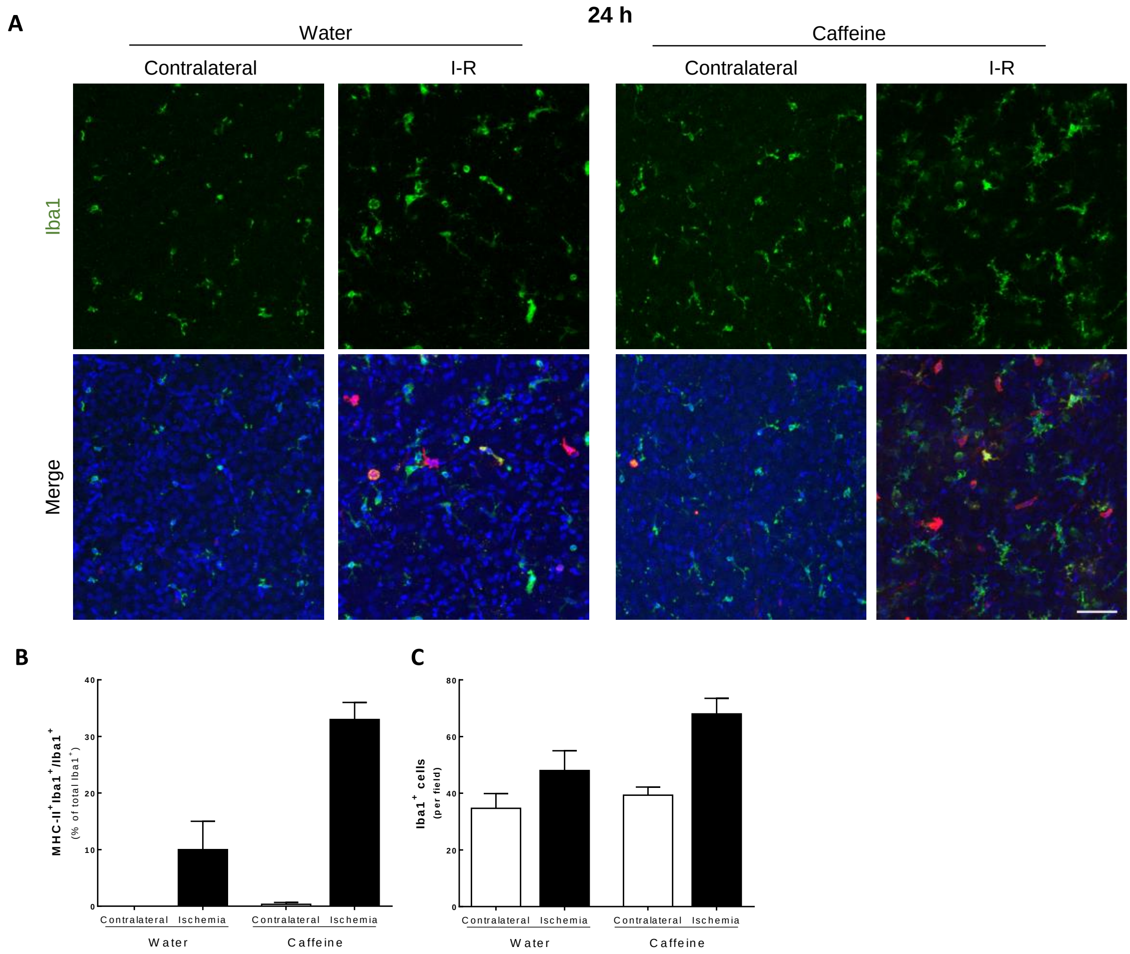

Supplementary Figure S2 - Effect of caffeine administration **(A, B, C)** in microglia reactivity elicited by transient ischemia at 24h of reperfusion. **A:** Microglia reactivity was assessed in retinal whole mounts by labeling with major histocompatibility complex class II (MHC-II, reactive microglia, red) and ionized calcium-binding adaptor molecule 1 (Iba1, general marker, green) at 24h of reperfusion. Nuclei were stained with DAPI (blue). Representative images for each condition are depicted. **B:** The number of activated microglia/macrophages (MHC-II$^+$Iba1$^+$ cells) was counted and expressed as percentage of total number of microglia/macrophages (Iba1$^+$ cells) in the retinal whole mounts at 24h of reperfusion. **C:** The number of microglia/macrophages (Iba1$^+$ cells) was counted in retinal whole mounts at 24h of reperfusion. Scale bar: 50 µm.

Supplementary Figure 3 - Retina whole mounts stained with anti-Iba1 in animals drinking caffeine at 7 days of reperfusion

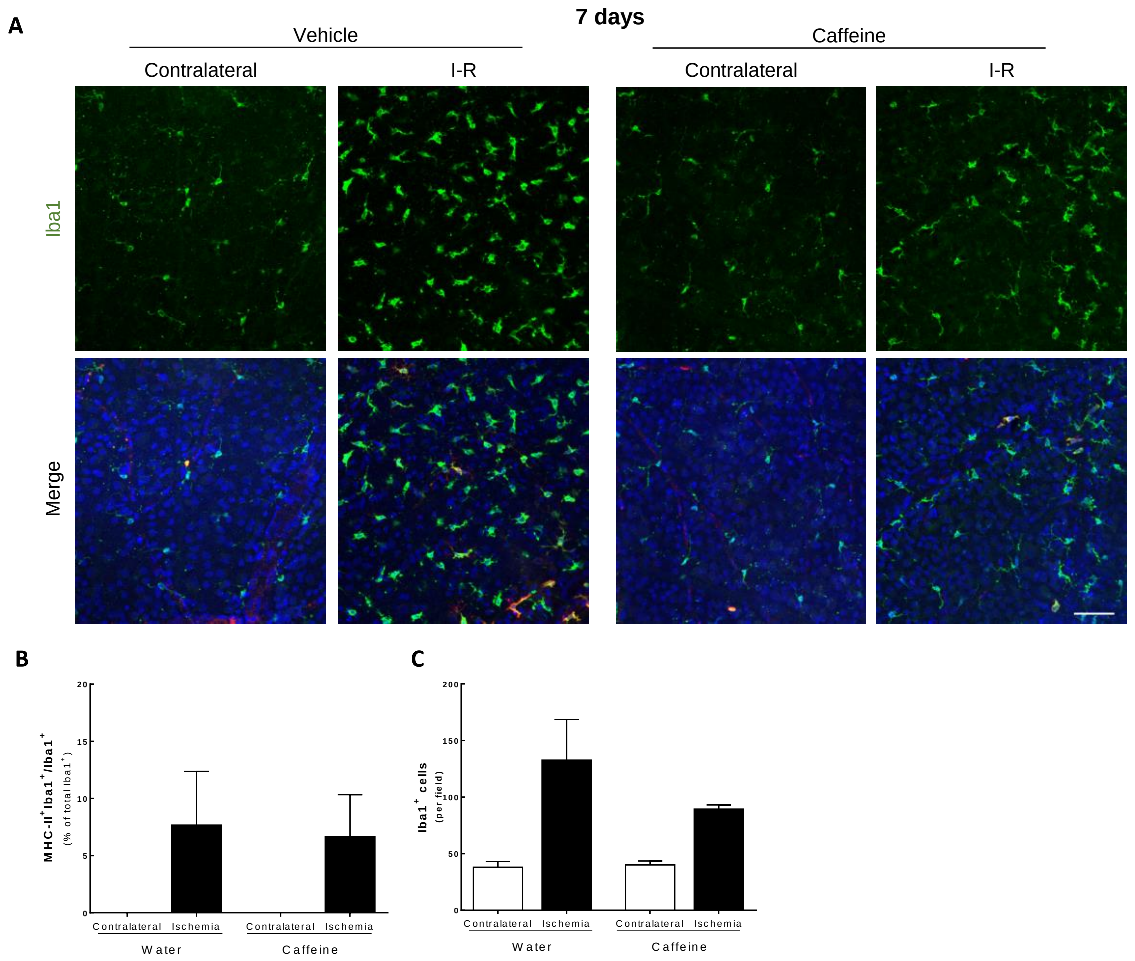

Supplementary Figure S3 - Effect of caffeine administration **(A, B, C)** in microglia reactivity elicited by transient ischemia at 7 days of reperfusion. **A:** Microglia reactivity was assessed in retinal whole mounts by labeling with major histocompatibility complex class II (MHC-II, reactive microglia, red) and ionized calcium-binding adaptor molecule 1 (Iba1, general marker, green) at 7 days of reperfusion. Nuclei were stained with DAPI (blue). Representative images for each condition are depicted. **B:** The number of activated microglia/macrophages (MHC-II^{+}Iba1^{+} cells) was counted and expressed as percentage of total number of microglia/macrophages (Iba1^{+} cells) in the retinal whole mounts at 7 days of reperfusion. **C:** The number of microglia/macrophages (Iba1^{+} cells) was counted in retinal whole mounts at 7 days of reperfusion. Scale bar: 50 µm.
